# Landscape Pattern Determines Neighborhood Size and Structure within a Lizard Population

**DOI:** 10.1371/journal.pone.0056856

**Published:** 2013-02-18

**Authors:** Wade A. Ryberg, Michael T. Hill, Charles W. Painter, Lee A. Fitzgerald

**Affiliations:** 1 Department of Wildlife and Fisheries Sciences, Biodiversity Research and Teaching Collections, College Station, Texas, United States of America; 2 Endangered Species Program, New Mexico Department of Game and Fish, Santa Fe, New Mexico, United States of America; University of Sydney, Australia

## Abstract

Although defining population structure according to discrete habitat patches is convenient for metapopulation theories, taking this approach may overlook structure within populations continuously distributed across landscapes. For example, landscape features within habitat patches direct the movement of organisms and define the density distribution of individuals, which can generate spatial structure and localized dynamics within populations as well as among them. Here, we use the neighborhood concept, which describes population structure relative to the scale of individual movements, to illustrate how localized dynamics within a population of lizards (*Sceloporus arenicolus*) arise in response to variation in landscape pattern within a continuous habitat patch. Our results emphasize links between individual movements at small scales and the emergence of spatial structure within populations which resembles metapopulation dynamics at larger scales. We conclude that population dynamics viewed in a landscape context must consider the explicit distribution and movement of individuals within continuous habitat as well as among habitat patches.

## Introduction

Populations of most species are spatially structured at multiple scales [1]–[3]. The scale of population structure often emerges as a consequence of the dispersal of individuals through heterogeneous landscapes [4], [5]. For example, when a species' dispersal capacity is limited relative to the spatial distribution of the habitat patches it can occupy, metapopulation structures can emerge, where “local” populations within habitat patches are connected regionally by infrequent dispersal among patches [6], [7]. Different metapopulation structures (e.g., core-satellite; [8]) can arise from the influence of habitat patch and landscape heterogeneity on the extinction and colonization of those local populations [9], [10].

Structure may also be found within populations occupying continuous habitats [2]. In particular, the spatial configuration of landscape features (i.e., landscape pattern) within continuous habitats can facilitate or constrain the movement of individuals and create spatial variation in population density [11]. This variation in population density can generate groups of strongly interacting individuals called “neighborhoods” that are organized regionally into continuous networks [12], [13]. The concept of a neighborhood, specifically the neighborhood size parameter, was devised by Wright [12] to approximate the effective size of a localized, randomly-mating unit within a continuously distributed population. In the context of identifying structure within populations, neighborhood size has intuitive appeal because it is calculated as the movement of a species relative to the density of individuals in the landscape and measures the contact-process of reproduction [14]. Thus, individual neighborhoods can have unique localized rates of recruitment as well as survivorship, and the persistence of the neighborhood network depends on dispersal or diffusion among neighborhoods just as in metapopulations [15]–[18]. As such, patterns of population structure traditionally conceptualized for metapopulation dynamics among habitat patches might therefore also emerge among networks of neighborhoods that are self-organized by landscape pattern within populations occupying continuous habitats.

**Figure 1:**
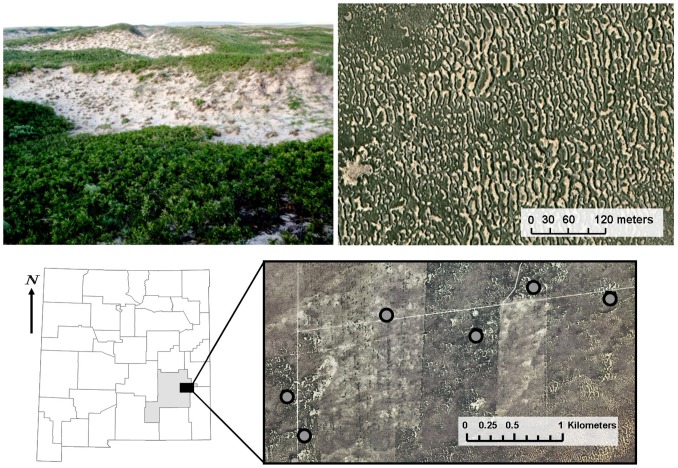
Study area, sampling sites, and landscape pattern. Sampling sites were located in Shinnery Oak sand-dune habitat found in Caprock Wildlife Area located in eastern Chaves County, New Mexico, U.S.A. Upper left picture shows a typical sand-dune blowout occupied by *Sceloporus arenicolus* in the foreground with many more blowouts in the background. Upper right picture from above illustrates how the form and arrangement of blowouts (brown) and Shinnery Oak matrix (green) can create variation in landscape pattern within continuous habitats. The effect of this variation in landscape pattern on localized lizard demography is unknown.

Here we predict that patterns of population structure thought to manifest regionally in metapopulations also occur at smaller scales within populations due to the response of individuals to landscape pattern within continuous habitats. We explore this prediction using multisite mark-recapture data from a population of the Dunes Sagebrush Lizard (*Sceloporus arenicolus*), an extreme habitat specialist occurring only in Shinnery Oak (*Quercus havardii*) sand-dune habitats of southeastern New Mexico and west Texas [19], [20]. Within continuous habitats, these lizards use wind-hollowed depressions, called sand-dune blowouts (Fig. 1). Individuals prefer larger and deeper blowouts with coarse sand-grain size, but they also readily move among interconnected blowouts of varying size [19]. These lizards do not occupy all blowouts, even those with seemingly suitable characteristics [21]. This observation suggests that landscape pattern might create structure within this population that resembles a metapopulation dynamic, because unoccupied blowouts may be colonized by lizards at some point in the future. Whether such structure is a result of the localized demographic responses of neighborhoods to landscape pattern must be empirically determined. Specifically, we evaluate whether the form and arrangement of blowouts creates a landscape pattern that may facilitate or constrain the movement of individual lizards, creating spatial variation in the density and therefore organization of lizards into neighborhoods with consequences for localized dynamics within lizard populations occupying continuous habitat.

## Methods

### Ethics statement

This study was carried out in strict accordance with recommendations in the Guidelines for use of Live Amphibians and Reptiles in Field Research compiled by the American Society of Ichthyologists and Herpetologists (ASIH), The Herpetologists' League (HL), and the Society for the Study of Amphibians and Reptiles (SSAR). Consistent with those recommendations, lizards were marked with toe-clips rather than PIT tags, which require the injection of large objects into relatively small reptiles. Toe-clipping caused only momentary pain and distress, and we rarely encountered any significant bleeding. When bleeding did occur, we applied pressure until the bleeding subsided and then applied triple antibiotic ointment on the wound. This protocol for wild capture and handling of lizards was approved by the Institutional Animal Care and Use Committee of Texas A&M University (Permit Number: AUP 7–159 and 2011–130). All field data were collected on New Mexico public lands. Permits for field work on this state threatened lizard species were approved by New Mexico Department of Game and Fish (Permit Number: 1755).

### Study site and data collection

We sampled lizards at Caprock Wildlife Area approximately 48 km east of Roswell, NM. In this area, we selected six sites located within a contiguous patch of Shinnery Oak sand-dune habitat occupied by *S. arenicolus* that varied in landscape pattern (Fig. 1, Supporting Information, Table S1). Previous genetic research failed to detect genetic population structure across this contiguous patch of habitat suggesting that lizards occupying the six sites were part of a single population [22]. Indeed, there was no evidence that variation in landscape pattern in this area created genetic structure among sand-dune blowouts at fine spatial scales. Thus, any observed differences in demographic rates between these sites varying in landscape pattern could not be attributed to genetic differences among lizards at each site. Pairwise distance between sites ranged from 0.6 to 3.6 km. To estimate localized dynamics contributing to neighborhood structure within this population, we designed a multisite mark-recapture study where pitfall trapping grids were constructed at each site. Grids consisted of 36 (20 L) buckets in a 6×6 pattern spaced 15 m apart. Thus, we sampled an area of 5,625 m^2^ at each site, which is large enough to contain dozens of lizard home ranges (mean size = 436 m^2^, Hill and Fitzgerald unpublished data).

We sampled lizards during the peak activity season [23] in June 2005, June-July in 2006, May-September in 2007–08, and April-September in 2009. We opened traps for one week during each month of sampling, which resulted in 16–19 trapping occasions across the six sites over the five-year study (Table S1). We measured snout-vent length (SVL) and mass for all lizards captured. We also determined sex and reproductive status of females by palpation. Finally, before releasing each lizard at site of capture, we gave each lizard a unique and permanent mark, by toe clipping, and noted the location of capture.

### Landscape pattern

We characterized landscape pattern across the six sites by mapping their locations on two digitized landcover layers in a GIS. One layer was a classification of vegetation types for sites at a 1-m resolution derived from Landsat Enhanced Thematic Mapper (ETM) satellite imagery and aerial photos [24]. The other layer was a classification of sand-dune blowouts derived directly from 1-m digitally rectified orthoquarterquads (DOQQ's) taken in 2004 using ArcInfo (Environmental Systems Research Institute 1999–2005b). We measured landcover composition and structure from 100×100 m plots framing each sampling grid (75 m/side) with an additional 12.5 m per side. We clipped these landcover plots from the two landcover layers and calculated landscape metrics using Program FRAGSTATS [25]. Only Blowout and Shinnery Oak landcover types occurred in plots. Because lizards occur in association with blowouts within Shinnery Oak sand-dunes [19], [26], we focused landscape calculations on the Blowout landcover type. We used multiple metrics to quantify blowout area, edge, shape, and connectivity within landscape plots surrounding each site (Table S2; [25]).

In addition to the two-dimensional measures of landscape pattern described above, we quantified three-dimensional measures of blowout heterogeneity that contribute to variation in landscape pattern and have been shown to be important for predicting habitat selection and presence-absence of the lizard at larger scales throughout its range [19], [21], [27]. Specifically, we quantified depth for all individual blowouts occurring within the landcover plots surrounding each grid site. Depth was calculated as the vertical distance from lowest point in a blowout to highest point of surrounding dunes. We also measured elevation, slope, aspect, soil compaction, and percent vegetative cover at each pitfall trap using a level, compass, soil penetrometer, vegetation quadrat, and handheld GPS unit (Garmin GPSMap 76CSx, altimeter resolution = 30.5 cm; Table S3). Variation in these variables characterized the surface topography of the landcover plot surrounding each site.

### Analysis

To estimate demographic parameters for each site, we aggregated the data set into monthly trapping occasions, which were then organized into encounter histories. In Program MARK [28], we used these encounter histories to estimate the following parameters: apparent survival probability (*s*; probability that a lizard survives from time *t* to *t*+1, given it is alive at time *t*), recapture probability (*p*; probability that a lizard alive at time *t* is captured at time *t*+1), recruitment rate (*f*; number of individuals recruited during the interval per member of the population alive at time *t*), rate of population change (λ), and population size (N) (Supporting Information, Protocol S1). We calculated lizard density by dividing estimates of N by the area (5,625 m^2^) of each grid site. We then used these estimates of lizard density across sites and site-specific measures of lizard movements collected from the subsequent recaptures of individual lizards within grids to calculate neighborhood size (NS) for each site using the equation below:
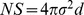
where *σ^2^* is the variance of intra-grid movements for individual lizards along a single axis (i.e., step lengths) and *d* is the density of individuals [12]. We were also interested in estimating probabilities of movement among sites (ψ; probability of moving to a site in which the marked individual may potentially be encountered, given it is alive and at that site); however, no marked individuals were detected moving among sites. As a result, we estimated unique demographic parameters for each of the six sites, and we restricted our analysis of lizard dispersal to estimates of site-specific diffusion rates (Protocol S1).

The diffusion rate (*D*) is a single metric of population spread that incorporates both the mean and variance in movement distances over time [29]. We calculated movement distances from the subsequent recaptures of individual lizards among pitfalls within each grid. After meeting the assumptions of environmental quasi-homogeneity and uncorrelated successive movements (Protocol S1), we characterized diffusion rates at each site using uncorrelated random walk procedures. The estimated diffusion rate for an uncorrelated random walk in two-dimensional space from *n* moves is:
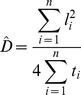
where *l_i_* is the length of the *i*-th move and *t_i_* is its duration [29]. We scaled the duration of movements by generation time of the lizard (i.e., m^2^/generation; generation time = 1 year; [19]). This total path-length re-scaling is common when estimating diffusion rates for territorial species that may move only once during their lifetime as juveniles.

We used linear regression to evaluate the relationships between 1) demographic rates (e.g., survival, recruitment) and variables describing landscape pattern, 2) demographic rates and neighborhood size, 3) neighborhood size and the variables describing landscape pattern, and 4) demographic rates and diffusion rates across sites.

## Results

We captured 1,463 lizards over the 5-year mark-recapture study, and 521 of those were recaptures. We used these recaptures to generate 303 encounter histories across all six sites. There were no movements of marked individuals between sites.

Using model selection procedures for estimates of apparent survival (*s*), we observed similarities in model structure across the six sites and determined that survival was high for lizards in this region ranging from 0.88 to 0.95 per trapping interval or 0.46 to 0.74 annually (Table S4). We were, however, unable to statistically distinguish estimates of survival between sites (Table S5). For recapture probability (*p*), we found support for constant model structures at sites 1, 3, 5 and 6 with estimates ranging from 0.21 to 0.31, and for time-dependent structures at sites 2 and 4 (Table S4), although time-dependent estimates of recapture probabilities were statistically indistinguishable (Table S6).

**Figure 2:**
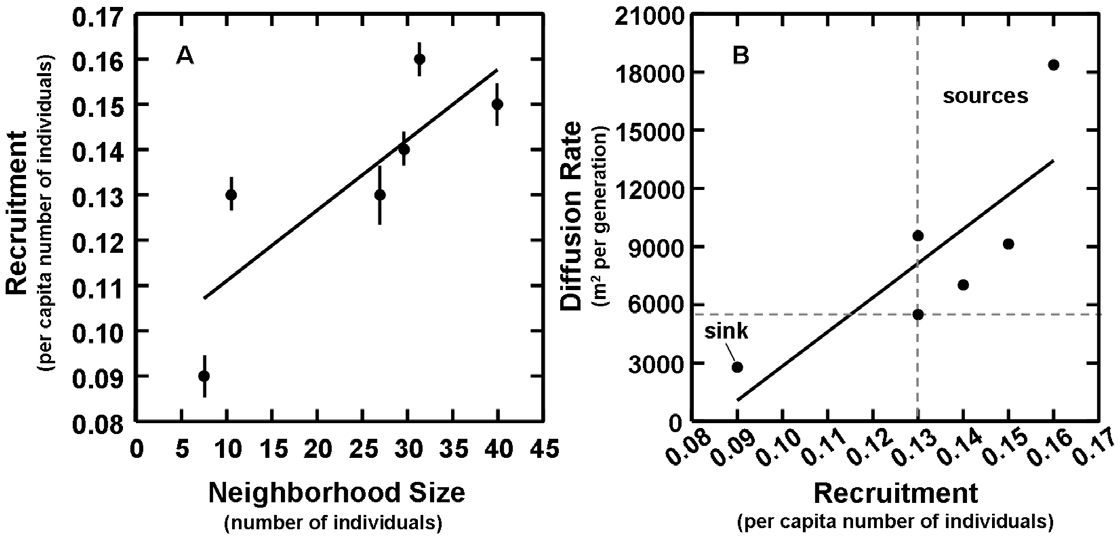
Neighborhood recruitment and diffusion. (A) Larger neighborhood sizes maintained significantly higher recruitment rates (R^2^ = 0.82, df = 4, P = 0.05; bars = ±SE), and (B) higher recruitment rates generated higher diffusion rates (R^2^ = 0.80, df = 4, P = 0.05). Dashed lines estimate the threshold levels of recruitment, 0.13 (vertical), and diffusion rate, 5,625 m^2^ (horizontal) required to balance population losses across sites and occupy the same area in the landscape (i.e., spatial equilibrium). Sites found above both thresholds (upper right) are identified as sources; the site found below both thresholds (lower left) is identified as a sink (see text).

For recruitment (*f*) and the rate of population change (λ), we observed time-dependent model structures across all sites except site 3, where data supported models with constant structures (Tables S7 and S8). However, post-hoc tests of synchrony (Supporting Information, Fig. S1) between time-dependent estimates of recruitment and population change within sites indicated that sites were demographically open with frequent lizard movements into and out of sites across monthly trapping intervals [30]. Because movements at such fine temporal scales likely included many lizard foraging forays into and out of the site rather than emigration or immigration among sites [31], and because we were interested in only spatial variation in demographic rates to identify structure within the population, we used time-invariant model estimates for recruitment and the rate of population change for each site. Time-invariant estimates of recruitment across sites ranged from 0.09 to 0.16 (Fig. 2A), whereas time-invariant estimates for the rate of population change across sites did not vary and were indistinguishable from 1.0 indicating demographic equilibrium.

From models of local population size, we observed estimates ranging from 30 to 144 individuals across sites, and we used these estimates to calculate lizard density, which ranged from 0.5 to 2.6 lizards per 100 m^2^ (Table 1). We also calculated mean step length, total path length, mean number of moves per path, and turning angle per move for each of the 303 lizards comprising the encounter histories (Table 2). Using estimated lizard density and the variance in step length from each site, we calculated neighborhood size, which ranged from 7.5 to 40 lizards across sites (Fig. 2A). From the movement components calculated above, we also estimated diffusion rates for each site, which ranged from 2,786 to 18,371 m^2^/generation (Table 2).

**Table 1:** Site specific model estimates of local population size (*N*) followed by estimated density per 100 m^2^ for *S. arenicolus* across 6 sites.

Site	*N*	SE	95% CI	Density
1	72	6	62–83	1.3
2	120	11	103–145	2.1
3	30	4	25–43	0.5
4	144	7	131–159	2.6
5	48	4	40–56	0.9
6	42	5	34–57	0.7

Also shown are the standard error (SE) and confidence interval (95% CI).

**Table 2:** Mean step length, total path length, number of moves, turning angles, and population-level diffusion rates for *S. arenicolus* across 6 sites.

Site	Step Length (m)	Total Path Length (m)	Number of Moves	Turning Angle									Diffusion Rate
				0	45	90	135	180	225	270	315	Sum	m^2^/gen
1	22.7 (0.6)	32.5 (5.2)	1.4 (0.2)	0	1	2	1	7	2	2	0	15	9,553
2	22.9 (1.1)	27.8 (3.1)	1.2 (0.1)	1	0	3	0	3	1	0	1	9	7,034
3	27.6 (4.2)	30.9 (6.4)	1.1 (0.1)	0	0	1	0	0	0	1	0	2	5,508
4	20.1 (0.7)	30.4 (2.8)	1.5 (0.1)	1	2	6	5	18	3	3	2	40	18,371
5	29.2 (2.6)	39.8 (7.3)	1.4 (0.2)	1	1	0	3	3	0	0	0	8	9,134
6	21.7 (1.5)	31.8 (5.1)	1.5 (0.2)	0	0	1	1	2	1	2	0	7	2,786

Standard errors (SE) are also shown.

Although recruitment was spatially variable, we observed no significant relationships between it and other spatially variable factors like lizard density, movement, or the different metrics describing landscape pattern (df = 4, *P*>0.1; Table 3). Instead, we found a significant positive relationship between recruitment and neighborhood size across sites (R^2^ = 0.82, df = 4, *P* = 0.05; Fig. 2A). We found significant positive relationships between neighborhood size and mean blowout contiguity, slope angle, east and west aspects, and the compactness of sand (Table 3). We also observed significant negative relationships between neighborhood size and variation in blowout area, contiguity, and the compactness of sand (Table 3). Finally, we observed a significant positive relationship between recruitment and diffusion rates across sites (R^2^ = 0.80, df = 4, *P* = 0.05; Fig. 2B).

**Table 3:** Regression coefficients for *S. arenicolus* neighborhood size and mean and coefficient of variation in Blowout landcover metrics (Table S2) and blowout habitat variables (Table S3) at 6 sites.

Landcover metric	Correlation with neighborhood size	
	Mean	Coefficient of Variation
Area	0.28	−0.76^*^
Perimeter	0.25	−0.66
Gyrate	0.10	−0.28
Shape	0.23	−0.35
Fractal	0.01	0.02
Circle	0.60	−0.22
Contiguity	0.79^*^	−0.82^**^
Isolation	0.52	0.55

Habitat variable	Mean	Coefficient of Variation
Depth	0.26	−0.34
Elevation	−0.12	0.43
Soil compaction	0.74^*^	−0.86^***^
Slope	0.83^**^	0.25
North-South facing	−0.61	0.41
East-West facing	0.49	0.76^*^
Cover	0.06	0.47

Significant relationships at α = 0.10, 0.05, and 0.025 are symbolized by ^*^, ^**^, and ^***^, respectively (df = 4).

Because neighborhoods with higher recruitment rates (Fig. 2A) were also expanding faster or contributing more individuals to the surrounding landscape (Fig. 2B), we sought to further characterize this spatial pattern of population dynamics within a source-sink framework [32]. Using site area (5,625 m^2^) as a diffusion threshold for source-sink identity (horizontal dashed line, Fig. 2B), we identified four sites (1, 2, 4, 5), as net exporters of individuals or sources (i.e., emigration (e) > immigration (i); *D*>5,625 m^2^), site 6 as a net importer of individuals or a sink (i.e., e<i; *D*<5,625 m^2^), and site 3 as neutral (5,508 m^2^∼5,625 m^2^). Because recruitment estimates do not separate contributions from both birth and immigration rates, we could not directly evaluate whether putative sources or sinks, identified as net exporters or importers of individuals, also exhibited birth rates greater than death rates (b>d), in the case of sources, or birth rates less than death rates (b<d), in the case of sinks. Instead, we used indirect methods to compare birth and death rates across sites based on the following logical arguments. First, by plotting a conservative estimate of lizard population losses (mortality and emigration = 1 - mean *s* [lower 95% CI] across sites; Table S5) together with recruitment, we identified the threshold of recruitment, 0.13, necessary to balance population losses across sites (λ∼1, vertical dashed line, Fig. 2B). For all sites above this threshold (i.e., to the right), we presumed births and immigration were greater than mortality and emigration (b+i>d+e) and the opposite for sites below (i.e., to the left; b+i<d+e). By comparing sites relative to these two thresholds (vertical and horizontal), we observed that sites found above both thresholds (upper right, Fig. 2B) satisfied the inequalities, e>i and b+i>d+e, and that sites found below both thresholds (lower left, Fig. 2B) satisfied the inequalities, e<i and b+i<d+e. With a single rearrangement (i.e., subtracting d and i from both sides of the latter inequality in both cases), we deduced that sites identified as sources based on diffusion rates alone (e>i) also maintained more births than deaths, and that the site identified as a sink (e<i) maintained fewer births than deaths. This pattern is consistent with source-sink population structures [32].

## Discussion

Our results illustrated how landscape pattern within continuous habitat influenced the spatial organization of individual lizards into neighborhoods of different sizes, whose localized dynamics shaped patterns of population structure across the landscape. Specifically, we found that the spatial configuration of blowouts within continuous habitats regulated the size of lizard neighborhoods (Table 3), which was positively related to recruitment (Fig. 2A). Moreover, larger neighborhoods with higher recruitment exhibited population diffusion rates exceeding the spatial extent of our sampling areas and were determined to be net exporters of individuals (Fig. 2B). In contrast, smaller neighborhoods with lower recruitment exhibited population diffusion rates that were much smaller than the spatial extent of our sampling areas; persistence of such neighborhoods is likely dependant on net import of individuals. Additionally, sites with neighborhoods identified as net exporters were shown to maintain more births than deaths, and sites with neighborhoods importing individuals were shown to maintain fewer births than deaths. This spatial pattern of neighborhood structure is closely aligned with the metapopulation concept of source-sink populations [32]. As such, our results converge on the conclusion that patterns of population structure traditionally described by metapopulation dynamics among populations occupying discrete habitat patches may also emerge among neighborhoods within populations occupying continuous habitats across the landscape.

This conclusion emphasizes the link between individual movements at small scales and the emergence of spatial population structure that can generate metapopulation dynamics at larger scales [33]. Traditionally, the approach to making individual movements relevant to metapopulation theories has been to aggregate movements up to the level of distinct habitat patches where population dynamics are most discrete [7], [34]. Conceptually that approach restricts our point of reference to broad-scale movements among discrete habitat patches that reflect migration among populations in a metapopulation [5]. Defining population structure according to discrete habitat patches, however, may overlook patterns of structure within populations [34], which have been shown to have dramatic effects on the dynamics of populations and metapopulations at landscape scales [18]. Because our study focused on structure within a population occupying continuous habitat, we approached the problem from the opposite perspective and were able to reduce population dynamics to the scale of individual movements through the neighborhood concept. Specifically, we illustrated that spatial variation in recruitment was indirectly influenced by landscape pattern within continuous habitat through the effects of different blowout configurations on the organization of lizards into neighborhoods of different sizes. The exact mechanism, however, by which the spatial configuration of blowouts shaped individual lizard movements and contributed to the organization of lizard neighborhoods remains a topic of future research. Indeed, we observed no direct, significant relationships between recruitment, movement, density, and any of the metrics describing landscape pattern that might suggest a causal pathway. We suspect that many other intrinsic and extrinsic factors, such as predation, competitors, and food resources also influence animal movements and are themselves influenced by landscape configuration, making the organization of lizard neighborhoods a complex phenomenon. Thus, while our results reveal the importance of landscape pattern for *S. arenicolus* population dynamics, future research should help disentangle the complexities of abiotic/biotic interactions as they affect lizard movements and neighborhood organization. Indeed, the localized neighborhood dynamics (i.e., recruitment) and regional neighborhood connectivity (i.e., immigration by diffusion) that generated the source-sink structure we observed are likely best characterized through an explicit understanding of how the spatial configuration of blowouts within Shinnery Oak sand-dune habitats, as well as other factors (e.g., presence of predators, refugia, basking sites, conspecifics), directed movements at the level of individual lizards.

As our empirical data illustrate how landscape pattern within continuous habitats determines the size of lizard neighborhoods and thus their recruitment rates, it follows that alterations to landscape pattern in these habitats could disrupt neighborhood dynamics. For example, anthropogenic habitat loss and fragmentation could redirect individual lizard movements and cause neighborhoods to be reconfigured with consequences for the structure and persistence of neighborhoods within the population at larger scales. Cell-based simulation models have explored such conservation issues and illustrated how changes in habitat configuration as it relates to individual movements can facilitate or have little effect on population persistence [16], [ but see 18]. Our empirical results, on the other hand, carry the implication that disturbance of regional neighborhood dynamics (e.g., emigration, immigration by diffusion) through the erosion of landscape pattern within continuous habitat could cause the localized extirpation of sink neighborhoods within the population and threaten population connectivity at larger scales. Mechanisms related to the concept of habitat degradation are much less understood than outright habitat loss and conversion, and we suggest disruption of neighborhood dynamics is potentially one such mechanism that could help explain population decline in degraded habitats.

Our study demonstrates that population processes in small-scale neighborhoods reflect those conceived for metapopulations. Our study thus identifies the neighborhood concept as a means to scale individual movements in the landscape up to the dispersal dynamics traditionally reserved for metapopulations. Indeed, this finding illustrates how landscape pattern can generate patterns of structure within populations and is therefore part of a larger synthesis between landscape ecology and metapopulation theory [7], [9], [10], [35]. This finding also calls attention to the importance of habitat conservation at multiple scales. The conservation of populations in a landscape context must consider the explicit distribution and movement of individuals in space within continuous habitats as well as among habitat patches. With a better understanding of diffusion within habitats and dispersal among them, future work can focus on disentangling the relative importance of movement among neighborhoods and populations at different scales, and identify which is most important for species persistence under different anthropogenic threats.

## Supporting Information

Figure S1 **Estimates of recruitment and lambda (rate of population change).** For the first month on the x-axis “M” = May. Shaded periods on x-axis correspond to trapping occasions. Sites 1–6 are represented by dash-dot, dash-dot-dot, long-dash, dotted, solid, and short-dash lines, respectively. (A) Variable recruitment rates (note log scale) were observed across sites 1–2 and 4–6, but not site 3. (B) All sites exhibited variation in lambda that was asynchronous across years and sites. No sites exhibited synchrony between recruitment and lambda that would suggest a positive correlation.
(TIF)

Table S1 **Locality, year of construction, and number of trapping occasions for 6 sites at Caprock Wildlife Area, NM.**
(DOC)

Table S2 **Mean (coefficient of variation) of Blowout landcover metrics (meters) for landcover plots surrounding each site calculated in FRAGSTATS.** Blowout counts were 45, 45, 21, 29, 47, and 65 for sites 1–6, respectively.
(DOC)

Table S3 **Means (coefficient of variation) of three-dimensional and habitat quality metrics measured at each pitfall trap across sites.** Blowout counts for the depth metric were 10, 8, 11, 19, 18, and 8 for sites 1–6, respectively.
(DOC)

Protocol S1 **Demographic parameter estimation.**
(DOC)

Table S4 **Model ranking of Cormack–Jolly–Seber (CJS) mark–recapture models estimating apparent survival (** ***s*** **) and recapture probability (** ***p*** **) for** ***S. arenicolus*** **across 6 sites from 2005–09.**
(DOC)

Table S5 **Model-averaged estimates of apparent survival for female and male** ***S. arenicolus*** **across 6 sites from 2005–09 derived using Cormack-Jolly-Seber (CJS) mark-recapture models.**
(DOC)

Table S6 **Sex-specific or time-variant (depending on model ranking) estimates of recapture probability for** ***S. arenicolus*** **across 6 sites.**
(DOC)

Table S7 **Model ranking of Pradel mark–recapture models estimating apparent survival (** ***s*** **), recapture probability (** ***p*** **), and recruitment (** ***f*** **) for** ***S. arenicolus*** **across 6 sites from 2005–09.**
(DOC)

Table S8 **Model ranking of Pradel mark–recapture models estimating apparent survival (** ***s*** **), recapture probability (** ***p*** **), and rate of population change (λ) for** ***S. arenicolus*** **across 6 sites from 2005–09.**
(DOC)
