# Supplementary figures and images for: Landscape Pattern Determines Neighborhood Size and Structure within a Lizard Population
Source: PLoS One. 2013 Feb 18;8(2):e56856. doi: 10.1371/journal.pone.0056856 (PMC3575499; doi:10.1371/journal.pone.0056856)

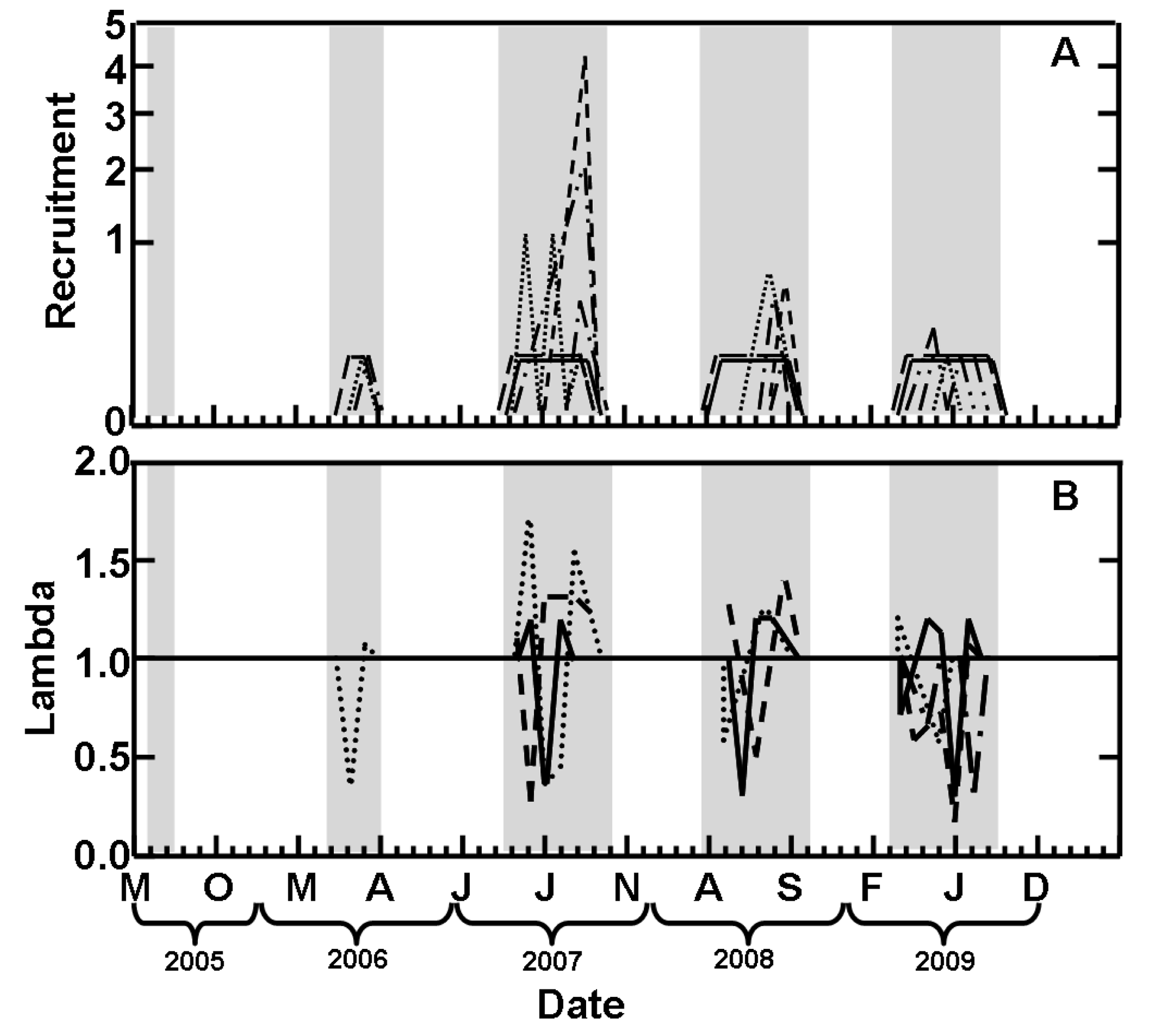

Supplement: Figure S1 — Estimates of recruitment and lambda (rate of population change). For the first month on the x-axis “M” = May. Shaded periods on x-axis correspond to trapping occasions. Sites 1–6 are represented by dash-dot, dash-dot-dot, long-dash, dotted, solid, and short-dash lines, respectively. (A) Variable recruitment rates (note log scale) were observed across sites 1–2 and 4–6, but not site 3. (B) All sites exhibited variation in lambda that was asynchronous across years and sites. No sites exhibited synchrony between recruitment and lambda that would suggest a positive correlation. (TIF) [file pone.0056856.s001.tif]
