# Supplementary material for: Landscape Pattern Determines Neighborhood Size and Structure within a Lizard Population
Source: PLoS One. 2013 Feb 18;8(2):e56856. doi: 10.1371/journal.pone.0056856 (PMC3575499; doi:10.1371/journal.pone.0056856)
Supplement: Table S1 — Locality, year of construction, and number of trapping occasions for 6 sites at Caprock Wildlife Area, NM. (DOC) [file pone.0056856.s002.doc]

Supporting Information

| Table S1. Locality, year of construction, and number of trapping occasions for 6 sites at Caprock Wildlife Area, NM. | | | |
| --- | --- | --- | --- |
| Site | Locality | Year Constructed | Trapping Occasions |
| 1 | 33° 27' 06'' N, 103° 47' 33'' W | 2005 | 19 |
| 2 | 33° 26' 50'' N, 103° 47' 56'' W | 2005 | 19 |
| 3 | 33° 27' 02'' N, 103° 47' 01'' W | 2006 | 18 |
| 4 | 33° 26' 30'' N, 103° 49' 13'' W | 2006 | 18 |
| 5 | 33° 26' 16'' N, 103° 49' 07'' W | 2007 | 16 |
| 6 | 33° 26' 57'' N, 103° 48' 32'' W | 2007 | 16 |
