# Supplementary material for: Landscape Pattern Determines Neighborhood Size and Structure within a Lizard Population
Source: PLoS One. 2013 Feb 18;8(2):e56856. doi: 10.1371/journal.pone.0056856 (PMC3575499; doi:10.1371/journal.pone.0056856)
Supplement: Table S2 — Mean (coefficient of variation) of Blowout landcover metrics (meters) for landcover plots surrounding each site calculated in FRAGSTATS. Blowout counts were 45, 45, 21, 29, 47, and 65 for sites 1–6, respectively. (DOC) [file pone.0056856.s003.doc]

| Table S2. Mean (coefficient of variation) of Blowout landcover metrics (meters) for landcover plots surrounding each site calculated in FRAGSTATS. Blowout counts were 45, 45, 21, 29, 47, and 65 for sites 1-6, respectively. | | | | | | | | |
| --- | --- | --- | --- | --- | --- | --- | --- | --- |
| Site | Area | Perimeter | Gyrate^*^ | Shape^†^ | Fractal^‡^ | Circle^§^ | Contiguity^**^ | Isolation^††^ |

| 1 | 55.6 (4.68) | 30.9 (3.10) | 1.8 (1.72) | 1.2 (0.39) | 1.2 (0.12) | 0.5 (0.27) | 0.3 (0.88) | 4.0 (0.46) |
| --- | --- | --- | --- | --- | --- | --- | --- | --- |
| 2 | 60.4 (4.25) | 34.7 (3.17) | 2.1 (2.14) | 1.2 (0.47) | 1.2 (0.12) | 0.5 (0.27) | 0.3 (0.91) | 4.8 (0.61) |
| 3 | 169.0 (3.37) | 69.1 (2.79) | 3.5 (2.25) | 1.4 (0.59) | 1.2 (0.13) | 0.5 (0.35) | 0.4 (0.83) | 3.5 (0.55) |
| 4 | 130.5 (4.66) | 54.5 (3.61) | 2.3 (2.07) | 1.3 (0.54) | 1.2 (0.10) | 0.5 (0.23) | 0.4 (0.79) | 4.4 (0.47) |
| 5 | 52.8 (2.90) | 34.2 (1.69) | 2.5 (1.38) | 1.3 (0.33) | 1.2 (0.14) | 0.6 (0.25) | 0.5 (0.61) | 3.9 (0.52) |
| 6 | 51.4 (5.15) | 36.9 (3.90) | 1.7 (2.12) | 1.3 (0.59) | 1.2 (0.13) | 0.5 (0.29) | 0.3 (1.01) | 3.0 (0.45) |

| ^*^mean radius of gyration; measures the average extent of blowouts from the blowout centroid |
| --- |
| ^†^mean shape index; measures mean blowout shape |
| ^‡^mean fractal dimension; measure of shape complexity (range 1-2 for shapes with simple to convoluted perimeters) |
| ^§^mean related circumscribing circle; measure of how compact blowouts are |
| ^**^mean contiguity index; measures cell contiguity within blowouts, boundary configuration and thus blowout shape |
| ^††^mean Euclidian nearest-neighbor distance; distance from reference blowout edge to nearest blowout edge |
