# Supplementary material for: Landscape Pattern Determines Neighborhood Size and Structure within a Lizard Population
Source: PLoS One. 2013 Feb 18;8(2):e56856. doi: 10.1371/journal.pone.0056856 (PMC3575499; doi:10.1371/journal.pone.0056856)
Supplement: Table S3 — Means (coefficient of variation) of three-dimensional and habitat quality metrics measured at each pitfall trap across sites. Blowout counts for the depth metric were 10, 8, 11, 19, 18, and 8 for sites 1–6, respectively. (DOC) [file pone.0056856.s004.doc]

| Table S3. Means (coefficient of variation) of three-dimensional and habitat quality metrics measured at each pitfall trap across sites. Blowout counts for the depth metric were 10, 8, 11, 19, 18, and 8 for sites 1-6, respectively. | | | | | | | |
| --- | --- | --- | --- | --- | --- | --- | --- |
| Site | Depth^*^ | Elevation (m) | Soil compaction^†^ | Slope^‡^ | North-South^§^ | East-West^**^ | Cover^††^ |
| 1 | 6.34 (0.361) | 1292.7 (0.001) | 2.59 (1.052) | 11.7 (0.715) | 0.08 (7.933) | -0.05 (-15.346) | 51.7 (0.55) |
| 2 | 9.19 (0.485) | 1292.8 (0.002) | 3.86 (0.828) | 12.6 (0.869) | 0.02 (30.82) | 0.20 (3.677) | 52.6 (0.47) |
| 3 | 7.45 (0.264) | 1298.5 (0.002) | 5.64 (0.528) | 11.8 (0.99) | -0.07 (-10.095) | -0.22 (-2.971) | 54.4 (0.40) |
| 4 | 6.60 (0.364) | 1270.3 (0.002) | 4.15 (0.696) | 14.0 (0.776) | 0.03 (25.663) | -0.14 (-5.106) | 58.7 (0.61) |
| 5 | 5.18 (0.415) | 1279.9 (0.001) | 4.06 (0.459) | 13.2 (0.712) | 0.04 (17.531) | 0.28 (2.498) | 34.6 (0.97) |
| 6 | 4.89 (0.575) | 1277.5 (0.001) | 1.53 (0.963) | 10.0 (0.723) | 0.14 (5.433) | -0.10 (-6.18) | 41.5 (0.57) |
| ^*^vertical distance (in meters) measured perpendicularly from lowest to highest points in each blowout | | | | | | | |
| ^†^soil compaction measured at a point 2 m from each pitfall trap | | | | | | | |
| ^‡^degrees from level along dominant slope within 1m^2^ frame measured at a point 2 m from each pitfall trap | | | | | | | |
| ^§^northern to southern aspect (in radians) taken along dominant slope | | | | | | | |
| ^**^eastern to western aspect (in radians) taken along dominant slope | | | | | | | |
| ^††^percent sand cover vs. vegetative structure within 1m^2^ frame measured at a point 2 m from each pitfall trap | | | | | | | |
