# Supplementary material for: Landscape Pattern Determines Neighborhood Size and Structure within a Lizard Population
Source: PLoS One. 2013 Feb 18;8(2):e56856. doi: 10.1371/journal.pone.0056856 (PMC3575499; doi:10.1371/journal.pone.0056856)
Supplement: Protocol S1 — Demographic parameter estimation. (DOC) [file pone.0056856.s005.doc]

Protocol S1

*Estimating demographic parameters*

For each demographic parameter, we developed *a priori* models for analysis, evaluated goodness-of-ﬁt, and estimated an overdispersion parameter (*ĉ*) for the data set. We used an information theoretic approach to select the most parsimonious model, based on the AICc model selection criterion (lower AICc values represented better fitting models) [1]. The AICc is the Akaike’s information criterion [2] corrected for small sample bias [3]. Finally, we estimated parameters from all candidate models [4,5] using a model-average approach that incorporated model selection uncertainty.

*Survival and recapture probability* - We used a Cormack–Jolly–Seber (CJS) open population model [6-8] to estimate apparent survival (*s*) and recapture probability (*p*) of lizards for each of the six sites. We examined sex-dependent characteristics and temporal variation of these parameters with models grouped by sex (g) and crossed with constant (.) or variable (*t*) time effects (16 models). The global model included estimates of sex and time effects plus the interaction between them for both apparent survival probability and recapture probability. For all six sites, we assessed goodness-of-fit with the global model using the median-*ĉ* approach in Program MARK. Estimates of *ĉ* ranged from 0.94 to 1.05 for all sites, which indicated no overdispersion and good ﬁt of data to the models. Consequently, we ran all analyses with estimates of *ĉ* = 1.00.

Using model selection procedures for estimates of apparent survival (*s*), we found similarities in model structure across the six sites with sex-dependent but constant trends for survival receiving the greatest support given the data (Table S4). Although there was some evidence of sex-dependent survival in model selection procedures, when considering the large 95% confidence intervals surrounding estimates of apparent survival, we were unable to differentiate survival between sexes and sites (Table S5). For estimates of recapture probability (*p*), we found support for sex-dependent but constant model structures at sites 1, 3, 5 and 6, and for time-dependent structures at sites 2 and 4 (Table S4). In those sites described by sex-dependent but constant structures, we observed estimates ranging from 0.21 to 0.31; however, given the large 95% confidence intervals in these estimates, we were unable to differentiate recapture probabilities between sexes and sites (Table S6). In sites exhibiting time-dependent model structures, we observed highly variable estimates for recapture probability through time; however, given the large 95% confidence intervals in these estimates, we were unable to distinguish recapture probabilities through time (Table S6).

*Recruitment and rate of population change* - We used models developed by Pradel [9] to estimate recruitment (*f*), which quantifies the number of additions to the population (i.e., births + immigration), and the rate of population change (λ), which determines whether populations were increasing (λ > 1.0), decreasing (λ < 1.0), or at equilibrium (λ = 1.0). We compared models where apparent survival probability, recapture probability, and recruitment or the rate of population change were either constant (.) or variable (*t*) (8 models per estimate of *f* and λ). Global models for each independent estimate of recruitment and rate of population change were fully time-dependent. For all six sites, we assessed goodness-of-fit of global models and estimated overdispersion (*ĉ* = χ^2^/df using combined χ^2^ values and degrees of freedom from tests 2 and 3) in Program RELEASE [6]. Estimates of *ĉ* ranged from 0.7 to 1.06 among sites for models of recruitment and rate of population change. We adjusted analyses for overdispersion where necessary.

For estimates of recruitment (*f*) and the rate of population change (λ), we found support for time-dependent model structures across all sites except for site 3, where data supported models with constant structures (Table S7 and S8). In sites exhibiting time-dependent model structures, we observed highly variable estimates for recruitment, which ranged from 0.03 to 4.20, and for the rate of population change, which ranged from 0.20 to 1.70 (Fig. S1).

*Post-hoc tests of synchrony* - When survival is constant and the rate of population change varies over time, then variation in recruitment is expected to mirror variation in the rate of population change unless there is emigration or immigration within sites [10]. As such, we conducted post-hoc tests to identify if there was synchrony between recruitment and the rate of population change within sites. After determining that there was no serial correlation in either of these time-dependent estimates (*f* or λ), we identified synchrony by measuring the tendency of the time-series for both estimates (*f* and λ) to move in the same direction within sites [11]. We found no evidence of synchrony between recruitment and the rate of population change. Of 82 consecutive trapping occasions where both recruitment and the rate of population change varied, we observed synchrony in only 1 interval (site 4 in May-June 2007; Fig. S1). In the remaining 17 consecutive trapping occasions, we observed no variation in either recruitment or the rate of population change. This asynchrony suggests that all sites were demographically open and movements into and out of sites were common across monthly trapping intervals [10]. Because movements at such fine temporal scales likely included many lizard foraging forays into and out of the site rather than emigration or immigration among sites [12], and because we were interested in only spatial variation in demographic rates within the population, we used time-invariant model estimates for recruitment and the rate of population change for each site (reported in main text).

*Population size* - We estimated local population size, N, across the six sites using the open-population POPAN parameterization [13] in Program MARK. In this analysis, we constrained variation in survival and capture rates over time based on estimates derived from CJS models above and because the same effort was used in all sampling occasions. We also treated *PENT* (probability of entrance) as time-dependent within the model, because having an equal fraction of the local population returning each sampling interval is unlikely biologically. Given these restrictions, we fitted a single model to estimate N across each site. We assessed goodness-of-fit and estimated overdispersion as indicated above. Across all six sites, estimates of *ĉ* ranged from 0.7 to 1.09. We adjusted analyses for overdispersion where necessary. We report the results of this analysis in the text.

*Assumptions of diffusion approaches*

Several assumptions must be made to estimate population spread using diffusion approaches. First, we assumed a quasi-homogeneous environment at each site, because the spatial scale of heterogeneity (e.g., mean distance between blowouts ~ 4 m; Table S2) was less than the scale of individual movements (e.g., mean step length > 20 m; Table 2). This observation indicates that lizards could move readily among adjacent blowouts within Shinnery Oak sand-dune habitat (i.e., their perceptual range is greater than the scale of environmental heterogeneity). Second, we assumed there was no directional persistence or any other correlation between successive movements (i.e., displacements were best described by an uncorrelated random walk). To validate this assumption, we conducted tests of independence among successive movements following the methods of Swihart and Slade [14]. Five of six sites exhibited no autocorrelation between successive movements (i.e., at *α*=0.05, all observed *t*^2^/*r*^2^ > critical values for upper bound, resulting in failure to reject the null hypothesis of independence) and one remaining site (3) contained insufficient movements to use the test. With no reason to suspect dependence among successive movements at only site 3 versus remaining sites, we characterized diffusion rates at each site using uncorrelated random walk procedures.

References in Protocol S1

1. Burnham KP, Anderson DR (2002) Model selection and multimodel inference: a practical information-theoretic approach. New York: Springer-Verlag. 488 p.

2. Akaike H (1973) Information theory as an extension of the maximum likelihood principle. In: Petrov BN, Csaki F, editors. Proceedings of the Second International Symposium on Information Theory. Budapest: Society Press. pp. 267–281.

3. Hurvich CM, Tsai CL (1989) Regression and time series model selection in small samples. Biometrika 76:297-307.

4. Lebreton, JD, Burnham KP, Clobert J, Anderson DR (1992) Modeling survival and testing biological hypotheses using marked animals: a uniﬁed approach with case studies. Ecol Monogr 62:67–118.

5. Anderson DR, Burnham KP, White GC (1994) AIC model selection in overdispersed capture–recapture data. Ecology 75:1780–1793.

6. Burnham KP, Anderson DR, White GC, Brownie C, Pollock PH (1987) Design and analysis methods for fish survival experiments based on release- recapture. Bethesda: American Fisheries Society Monograph No. 5. 737 p.

7. Pollock KH, Nichols JD, Brownie C, Hines JE (1990) Statistical inference for capture–recapture experiments. Wild Monogr 107:1–97.

8. Franklin AB, Anderson DR, Forsman ED, Burnham KP, Wagner FW (1996) Methods for collecting and analyzing demographic data on the Northern Spotted Owl. Studies in Avian Biology 17:12-20.

9. Pradel R (1996) Utilization of capture–mark–recapture for the study of recruitment and population growth rate. Biometrics 52:703–709.

10. Franklin AB (1992) Population regulation in Northern Spotted Owls: theoretical implications for management. In: McCullough DR, Barrett RH, editors. Wildlife 2001: Populations. Essex: Elsevier Science Publishers. pp. 815-827.

11. Buonaccorsi JP, Elkinton JS, Evans SR, Liebhold AM (2001) Measuring and testing for spatial synchrony. Ecology 82:1668–79.

12. Diez JM, Giladi I (2011) Scale-dependence of habitat sources and sinks. In: Liu J, Hull [V](http://www.lavoisier.fr/livre/resultats.asp?texte=HULL Vanessa&type_recherche=5), Morzillo AT, Wiens JA, editors. Sources, sinks and sustainability. Cambridge: Cambridge University Press. pp. 291-316.

13. Schwarz CJ, Arnason AN (1996) A general methodology for the analysis of open-model capture recapture experiments. Biometrics 52:860-873.

14. Swihart RK, Slade NA (1985) Testing for independence of observations in animal movements. Ecology 66:1176–1184.
