# Supplementary material for: Landscape Pattern Determines Neighborhood Size and Structure within a Lizard Population
Source: PLoS One. 2013 Feb 18;8(2):e56856. doi: 10.1371/journal.pone.0056856 (PMC3575499; doi:10.1371/journal.pone.0056856)
Supplement: Table S4 — Model ranking of Cormack–Jolly–Seber (CJS) mark–recapture models estimating apparent survival ( s ) and recapture probability ( p ) for S. arenicolus across 6 sites from 2005–09. (DOC) [file pone.0056856.s006.doc]

| Table S4. Model ranking of Cormack–Jolly–Seber (CJS) mark–recapture models estimating apparent survival (*s*) and recapture probability (*p*) for *Sceloporus arenicolus* across 6 sites from 2005-09. Shown are delta Akaike’s information criteria corrected for small sample size (ΔAICc), the AICc weight (AICc wt), the number of parameters and the deviance for each model. A ‘(·)’ denotes time-invariant parameters, ‘(*t*)’ denotes time-variant parameters, and ‘(*g*)’ denotes sex-dependent parameters. | | | | | |
| --- | --- | --- | --- | --- | --- |
| Site | Model | ΔAICc | AICc wt | Parameters | Deviance |
| 1 | *s*(.) *p*(.) | 0.00 | 0.49 | 2 | 229.7 |
|  | *s*(.) *p*(*g*) | 1.73 | 0.20 | 3 | 229.3 |
|  | *s*(*g*) *p*(.) | 1.74 | 0.20 | 3 | 229.3 |
|  | *s*(*g*) *p*(*g*) | 3.08 | 0.10 | 4 | 228.5 |
|  | *s*(.) *p*(*t*) | 16.28 | <0.01 | 19 | 202.5 |
| 2 | *s*(.) *p*(*t*) | 0.00 | 0.78 | 19 | 255.2 |
|  | *s*(*g*) *p*(*t*) | 2.78 | 0.19 | 20 | 255.2 |
|  | *s*(.) *p*(.) | 8.49 | 0.01 | 2 | 304.4 |
|  | *s*(.) *p*(*g*) | 9.38 | 0.01 | 3 | 303.2 |
|  | *s*(*g*) *p*(.) | 10.58 | <0.01 | 3 | 304.4 |
| 3 | *s*(.) *p*(.) | 0.00 | 0.31 | 2 | 100.2 |
|  | *s*(*g*) *p*(.) | 0.03 | 0.31 | 3 | 97.8 |
|  | *s*(*g*) *p*(*g*) | 0.39 | 0.25 | 4 | 95.7 |
|  | *s*(.) *p*(*g*) | 1.94 | 0.12 | 3 | 99.7 |
|  | *s*(*t*) *p*(.) | 39.83 | <0.01 | 18 | 74.1 |
| 4 | *s*(.) *p*(*t*) | 0.00 | 0.75 | 18 | 428.1 |
|  | *s*(*g*) *p*(*t*) | 2.23 | 0.25 | 19 | 428.0 |
|  | *s*(.) *p*(*g*t*) | 12.31 | <0.01 | 35 | 397.4 |
|  | *s*(*g*) *p*(*g*t*) | 14.30 | <0.01 | 36 | 396.7 |
|  | *s*(.) *p*(.) | 21.13 | <0.01 | 2 | 484.2 |
| 5 | *s*(.) *p*(.) | 0.00 | 0.55 | 2 | 147.6 |
|  | *s*(*g*) *p*(.) | 2.08 | 0.19 | 3 | 147.5 |
|  | *s*(.) *p*(*g*) | 2.16 | 0.19 | 3 | 147.6 |
|  | *s*(*g*) *p*(*g*) | 4.24 | 0.07 | 4 | 147.4 |
|  | *s*(*t*) *p*(.) | 18.87 | <0.01 | 15 | 131.8 |
| 6 | *s*(*g*) *p*(.) | 0.00 | 0.31 | 3 | 145.7 |
|  | *s*(.) *p*(.) | 0.25 | 0.27 | 2 | 148.1 |
|  | *s*(.) *p*(*g*) | 0.26 | 0.27 | 3 | 145.9 |
|  | *s*(*g*) *p*(*g*) | 1.54 | 0.14 | 4 | 144.9 |
|  | *s*(*t*) *p*(.) | 13.87 | <0.01 | 16 | 121.9 |
