# Supplementary material for: Landscape Pattern Determines Neighborhood Size and Structure within a Lizard Population
Source: PLoS One. 2013 Feb 18;8(2):e56856. doi: 10.1371/journal.pone.0056856 (PMC3575499; doi:10.1371/journal.pone.0056856)
Supplement: Table S5 — Model-averaged estimates of apparent survival for female and male S. arenicolus across 6 sites from 2005–09 derived using Cormack-Jolly-Seber (CJS) mark-recapture models. (DOC) [file pone.0056856.s007.doc]

| Table S5. Model-averaged estimates of apparent survival for female and male *Sceloporus arenicolus* across 6 sites from 2005-09 derived using Cormack-Jolly-Seber (CJS) mark-recapture models. Also shown are the standard error (SE), unconditional SE (USE), and 95% confidence intervals (CI) for each model-averaged estimate. | | | | | | |
| --- | --- | --- | --- | --- | --- | --- |
| Site | Sex | *s* | SE | USE | 95% CI |  |
| 1 | Female | 0.93 | 0.016 | 0.016 | 0.89-0.96 |  |
|  | Male | 0.93 | 0.018 | 0.019 | 0.88-0.96 |  |
| 2 | Female | 0.95 | 0.010 | 0.010 | 0.93-0.97 |  |
|  | Male | 0.95 | 0.010 | 0.010 | 0.93-0.97 |  |
| 3 | Female | 0.94 | 0.025 | 0.029 | 0.85-0.98 |  |
|  | Male | 0.88 | 0.044 | 0.060 | 0.71-0.96 |  |
| 4 | Female | 0.94 | 0.010 | 0.010 | 0.92-0.96 |  |
|  | Male | 0.94 | 0.010 | 0.010 | 0.92-0.96 |  |
| 5 | Female | 0.93 | 0.023 | 0.023 | 0.87-0.96 |  |
|  | Male | 0.93 | 0.024 | 0.025 | 0.86-0.96 |  |
| 6 | Female | 0.93 | 0.027 | 0.032 | 0.83-0.97 |  |
|  | Male | 0.95 | 0.020 | 0.023 | 0.88-0.98 |  |
