# Supplementary material for: Landscape Pattern Determines Neighborhood Size and Structure within a Lizard Population
Source: PLoS One. 2013 Feb 18;8(2):e56856. doi: 10.1371/journal.pone.0056856 (PMC3575499; doi:10.1371/journal.pone.0056856)
Supplement: Table S6 — Sex-specific or time-variant (depending on model ranking) estimates of recapture probability for S. arenicolus across 6 sites. (DOC) [file pone.0056856.s008.doc]

| Table S6. Sex-specific or time-variant (depending on model ranking) estimates of recapture probability for *S. arenicolus* across 6 sites. Also shown are standard error (SE), unconditional SE (USE) and 95% confidence intervals (CI) for model-averaged estimates. | | | | | |
| --- | --- | --- | --- | --- | --- |
| Site | Sex/Interval | *p* | SE | USE | 95% CI |
| 1 | Female | 0.27 | 0.042 | 0.043 | 0.19-0.36 |
|  | Male | 0.29 | 0.049 | 0.054 | 0.19-0.40 |
| 2 | June 2005-06 | 0.66 | 0.18 | 0.20 | 0.26-0.91 |
|  | June-July (2006) | 0.20 | 0.08 | 0.08 | 0.09-0.41 |
|  | July-May (2007) | 0.59 | 0.12 | 0.14 | 0.31-0.81 |
|  | May-June (2007) | 0.13 | 0.07 | 0.07 | 0.04-0.33 |
|  | June-July (2007) | 0.09 | 0.06 | 0.06 | 0.02-0.30 |
|  | July-Aug (2007) | 0.05 | 0.05 | 0.05 | 0.01-0.30 |
|  | Aug-Sept (2007) | 0.005 | 0.001 | 0.03 | 0.001-0.06 |
|  | Sept-May (2008) | 0.37 | 0.13 | 0.13 | 0.16-0.64 |
|  | May-June (2008) | 0.13 | 0.07 | 0.07 | 0.04-0.34 |
|  | June-July (2008) | 0.18 | 0.08 | 0.08 | 0.07-0.40 |
|  | July-Aug (2008) | 0.05 | 0.04 | 0.05 | 0.006-0.29 |
|  | Aug-Sept (2008) | 0.13 | 0.07 | 0.07 | 0.04-0.35 |
|  | Sept-April (2009) | 0.19 | 0.10 | 0.10 | 0.06-0.47 |
|  | April-May (2009) | 0.27 | 0.10 | 0.10 | 0.12-0.51 |
|  | May-June (2009) | 0.19 | 0.09 | 0.09 | 0.07-0.43 |
|  | June-July (2009) | 0.24 | 0.10 | 0.10 | 0.10-0.49 |
|  | July-Aug (2009) | 0.05 | 0.05 | 0.05 | 0.006-0.31 |
|  | Aug-Sept (2009) | 0.25 | 0.09 | 0.10 | 0.11-0.47 |
| 3 | Female | 0.21 | 0.06 | 0.07 | 0.10-0.38 |
|  | Male | 0.27 | 0.08 | 0.10 | 0.12-0.50 |
| 4 | June-July (2006) | 0.60 | 0.14 | 0.14 | 0.33-0.83 |
|  | July-May (2007) | 0.69 | 0.23 | 0.23 | 0.21-0.95 |
|  | May-June (2007) | 0.25 | 0.13 | 0.13 | 0.08-0.56 |
|  | June-July (2007) | 0.38 | 0.11 | 0.11 | 0.19-0.60 |
|  | July-Aug (2007) | 0.0002 | 0.00002 | 0.00003 | 0.00002-0.005 |
|  | Aug-Sept (2007) | 0.32 | 0.10 | 0.10 | 0.15-0.54 |
|  | Sept-May (2008) | 0.61 | 0.12 | 0.12 | 0.37-0.80 |
|  | May-June (2008) | 0.46 | 0.08 | 0.08 | 0.31-0.62 |
|  | June-July (2008) | 0.17 | 0.06 | 0.06 | 0.08-0.32 |
|  | July-Aug (2008) | 0.18 | 0.06 | 0.06 | 0.09-0.33 |
|  | Aug-Sept (2008) | 0.17 | 0.06 | 0.06 | 0.08-0.33 |
|  | Sept-April (2009) | 0.23 | 0.07 | 0.07 | 0.12-0.41 |
|  | April-May (2009) | 0.40 | 0.08 | 0.09 | 0.25-0.57 |
|  | May-June (2009) | 0.38 | 0.08 | 0.08 | 0.24-0.54 |
|  | June-July (2009) | 0.35 | 0.08 | 0.08 | 0.21-0.51 |
|  | July-Aug (2009) | 0.15 | 0.06 | 0.06 | 0.07-0.30 |
|  | Aug-Sept (2009) | 0.17 | 0.06 | 0.12 | 0.04-0.53 |
| 5 | Female | 0.30 | 0.05 | 0.05 | 0.21-0.42 |
|  | Male | 0.31 | 0.06 | 0.06 | 0.21-0.43 |
| 6 | Female | 0.23 | 0.05 | 0.06 | 0.13-0.37 |
|  | Male | 0.28 | 0.06 | 0.07 | 0.17-0.42 |
