# Supplementary material for: Landscape Pattern Determines Neighborhood Size and Structure within a Lizard Population
Source: PLoS One. 2013 Feb 18;8(2):e56856. doi: 10.1371/journal.pone.0056856 (PMC3575499; doi:10.1371/journal.pone.0056856)
Supplement: Table S7 — Model ranking of Pradel mark–recapture models estimating apparent survival ( s ), recapture probability ( p ), and recruitment ( f ) for S. arenicolus across 6 sites from 2005–09. (DOC) [file pone.0056856.s009.doc]

| Table S7. Model ranking of Pradel mark–recapture models estimating apparent survival (*s*), recapture probability (*p*), and recruitment (*f*) for *Sceloporus arenicolus* across 6 sites from 2005-09. Shown are delta Akaike’s information criteria corrected for small sample size (ΔAICc), the AICc weight (AICc wt), the number of parameters and the deviance for each model. A ‘(·)’ denotes time-invariant parameters, ‘(*t*)’ denotes time-variant parameters. | | | | | |
| --- | --- | --- | --- | --- | --- |
| Site | Model | ΔAICc | AICc wt | Parameters | Deviance |
| 1 | *s*(.) *p*(*t*) *f*(*t*) | 0.00 | 0.71 | 26 | 176.6 |
|  | *s*(.) *p*(*t*) *f*(.) | 3.03 | 0.16 | 21 | 196.6 |
|  | *s*(.) *p*(.) *f*(.) | 3.67 | 0.11 | 3 | 244.3 |
|  | *s*(.) *p*(.) *f*(*t*) | 7.11 | 0.02 | 13 | 223.9 |
|  | *s*(*t*) *p*(.) *f*(.) | 22.9 | <0.01 | 20 | 219.7 |
| 2 | *s*(.) *p*(*t*) *f*(*t*) | 0.00 | 0.95 | 24 | 222.0 |
|  | *s*(.) *p*(.) *f*(*t*) | 6.61 | 0.03 | 8 | 272.2 |
|  | *s*(.) *p*(*t*) *f*(.) | 7.76 | 0.01 | 20 | 236.3 |
|  | *s*(.) *p*(.) *f*(.) | 25.7 | <0.01 | 3 | 298.8 |
|  | *s*(*t*) *p*(.) *f*(*t*) | 31.0 | <0.01 | 15 | 313.5 |
| 3 | *s*(.) *p*(.) *f*(.) | 0.00 | 0.95 | 3 | 87.7 |
|  | *s*(*t*) *p*(.) *f*(.) | 5.88 | 0.05 | 9 | 111.1 |
|  | *s*(.) *p*(.) *f*(*t*) | 21.0 | <0.01 | 10 | 105.1 |
|  | *s*(*t*) *p*(.) *f*(*t*) | 34.7 | 0.00 | 18 | 76.5 |
|  | *s*(.) *p*(*t*) *f*(.) | 46.8 | 0.00 | 16 | 102.0 |
| 4 | *s*(.) *p*(*t*) *f*(*t*) | 0.00 | 1.00 | 26 | 446.1 |
|  | *s*(*t*) *p*(*t*) *f*(*t*) | 26.7 | 0.00 | 42 | 429.8 |
|  | *s*(.) *p*(*t*) *f*(.) | 33.2 | 0.00 | 20 | 493.9 |
|  | *s*(*t*) *p*(.) *f*(*t*) | 53.3 | 0.00 | 28 | 494.4 |
|  | *s*(*t*) *p*(*t*) *f*(.) | 56.0 | 0.00 | 36 | 476.0 |
| 5 | *s*(.) *p*(.) *f*(*t*) | 0.00 | 0.99 | 9 | 183.0 |
|  | *s*(*t*) *p*(.) *f*(*t*) | 20.3 | <0.01 | 12 | 211.5 |
|  | *s*(.) *p*(.) *f*(.) | 23.8 | <0.01 | 3 | 229.3 |
|  | *s*(.) *p*(*t*) *f*(*t*) | 25.2 | 0.00 | 20 | 182.2 |
|  | *s*(.) *p*(*t*) *f*(.) | 29.8 | 0.00 | 17 | 197.4 |
| 6 | *s*(.) *p*(.) *f*(*t*) | 0.00 | 0.45 | 5 | 151.0 |
|  | *s*(.) *p*(*t*) *f*(*t*) | 0.18 | 0.41 | 16 | 118.2 |
|  | *s*(*t*) *p*(*t*) *f*(*t*) | 2.50 | 0.13 | 20 | 104.1 |
|  | *s*(*t*) *p*(.) *f*(.) | 7.50 | 0.01 | 9 | 148.1 |
|  | *s*(*t*) *p*(*t*) *f*(.) | 21.6 | <0.01 | 20 | 123.2 |
