# Supplementary material for: Landscape Pattern Determines Neighborhood Size and Structure within a Lizard Population
Source: PLoS One. 2013 Feb 18;8(2):e56856. doi: 10.1371/journal.pone.0056856 (PMC3575499; doi:10.1371/journal.pone.0056856)
Supplement: Table S8 — Model ranking of Pradel mark–recapture models estimating apparent survival ( s ), recapture probability ( p ), and rate of population change (λ) for S. arenicolus across 6 sites from 2005–09. (DOC) [file pone.0056856.s010.doc]

| Table S8. Model ranking of Pradel mark–recapture models estimating apparent survival (*s*), recapture probability (*p*), and rate of population change (λ) for *Sceloporus arenicolus* across 6 sites from 2005-09. Shown are delta Akaike’s information criteria corrected for small sample size (ΔAICc), the AICc weight (AICc wt), the number of parameters and the deviance for each model. A ‘(·)’ denotes time-invariant parameters, ‘(*t*)’ denotes time-variant parameters. | | | | | |
| --- | --- | --- | --- | --- | --- |
| Site | Model | ΔAICc | AICc wt | Parameters | Deviance |
| 1 | *s*(.) *p*(.) λ(*t*) | 0.00 | 0.99 | 17 | 127.2 |
|  | *s*(.) *p*(*t*) λ(*t*) | 8.88 | 0.01 | 38 | 165.2 |
|  | *s*(.) *p*(*t*) λ(.) | 46.1 | <0.01 | 21 | 122.0 |
|  | *s*(.) *p*(.) λ(.) | 52.5 | <0.01 | 3 | 196.6 |
|  | *s*(*t*) *p*(.) λ(*t*) | 53.1 | <0.01 | 26 | 244.3 |
| 2 | *s*(.) *p*(.) λ(*t*) | 0.00 | 1.00 | 17 | 165.2 |
|  | *s*(*t*) *p*(.) λ(*t*) | 30.4 | 0.00 | 24 | 215.2 |
|  | *s*(*t*) *p*(*t*) λ(.) | 73.6 | 0.00 | 29 | 223.3 |
|  | *s*(.) *p*(*t*) λ(.) | 82.5 | 0.00 | 21 | 256.5 |
|  | *s*(*t*) *p*(.) λ(.) | 96.0 | 0.00 | 10 | 298.2 |
| 3 | *s*(.) *p*(.) λ(*t*) | 0.00 | 0.66 | 14 | 70.9 |
|  | *s*(.) *p*(.) λ(.) | 1.38 | 0.33 | 3 | 111.1 |
|  | *s*(*t*) *p*(.) λ(*t*) | 10.6 | <0.01 | 20 | 40.4 |
|  | *s*(.) *p*(*t*) λ(.) | 42.1 | 0.00 | 18 | 88.4 |
|  | *s*(*t*) *p*(*t*) λ(.) | 42.5 | 0.00 | 21 | 62.6 |
| 4 | *s*(.) *p*(.) λ(*t*) | 0.00 | 1.00 | 18 | 436.7 |
|  | *s*(*t*) *p*(*t*) λ(.) | 38.0 | 0.00 | 28 | 450.4 |
|  | *s*(.) *p*(*t*) λ(.) | 61.9 | 0.00 | 20 | 493.9 |
|  | *s*(*t*) *p*(.) λ(.) | 97.6 | 0.00 | 12 | 547.9 |
|  | *s*(.) *p*(.) λ(.) | 113.2 | 0.00 | 3 | 582.8 |
| 5 | *s*(.) *p*(*t*) λ(*t*) | 0.00 | 0.97 | 30 | 63.5 |
|  | *s*(.) *p*(.) λ(*t*) | 6.79 | 0.03 | 13 | 138.2 |
|  | *s*(*t*) *p*(.) λ(.) | 50.7 | 0.00 | 7 | 198.2 |
|  | *s*(*t*) *p*(*t*) λ(.) | 54.6 | 0.00 | 20 | 162.9 |
|  | *s*(.) *p*(.) λ(.) | 72.5 | 0.00 | 3 | 229.3 |
| 6 | *s*(.) *p*(.) λ(*t*) | 0.00 | 1.00 | 11 | 74.8 |
|  | *s*(*t*) *p*(*t*) λ(.) | 69.1 | 0.00 | 18 | 119.3 |
|  | *s*(.) *p*(*t*) λ(.) | 73.8 | 0.00 | 15 | 135.5 |
|  | *s*(*t*) *p*(.) λ(.) | 75.6 | 0.00 | 10 | 153.4 |
|  | *s*(.) *p*(.) λ(.) | 81.8 | 0.00 | 3 | 177.5 |
